# A reverse dot blot assay for the screening of twenty mutations in four genes associated with NSHL in a Chinese population

**DOI:** 10.1371/journal.pone.0177196

**Published:** 2017-05-15

**Authors:** Siping Li, Qi Peng, Shengyun Liao, Wenrui Li, Qiang Ma, Xiaomei Lu

**Affiliations:** 1 Department of Laboratory, Dongguan Children's Hospital, Dongguan, Guangdong, China; 2 Department of Medical and Molecular Genetics, Dongguan Institute of Pediatrics, Dongguan, Guangdong, China; 3 Shenzhen Yilifang Biotech CO., LTD., Shenzhen, China; Hebrew University Hadassah Medical School, ISRAEL

## Abstract

**Background:**

Congenital deafness is one of the most distressing disorders affecting humanity and exhibits a high incidence worldwide. Most cases of congenital deafness in the Chinese population are caused by defects in a limited number of genes. A convenient and reliable method for detecting common deafness-related gene mutations in the Chinese population is required.

**Methods:**

We developed a PCR-reverse dot blot (RDB) assay for screening 20 hotspot mutations of *GJB2*, *GJB3*, *SLC26A4*, and *MT-RNR1*, which are common non-syndromic hearing loss (NSHL)–associated genes in the Chinese population. The PCR-RDB assay consists of multiplex PCR amplifications of 10 fragments in the target sequence of the four above-mentioned genes in wild-type and mutant genomic DNA samples followed by hybridization to a test strip containing allele-specific oligonucleotide probes. We applied our method to a set of 225 neonates with deafness gene mutations and 30 normal neonates.

**Results:**

The test was validated through direct sequencing in a blinded study with 100% concordance.

**Conclusions:**

The results demonstrated that our reverse dot blot assay is a reliable and effective genetic screening method for identifying carriers and individuals with NSHL among the Chinese population.

## Introduction

Hearing loss, which severely affects patients’ daily quality of life, is one of the most common sensory impairments, affecting approximately one to three newborns per every 1,000 live births[1, 2]. In China, there are approximately 0.8 million children (less than 7 years of age) with hearing impairments, and this proportion exhibits an annual increase of 30,000 children[3]. Hearing loss is etiologically heterogeneous, and it has been estimated that at least two thirds of the cases of childhood-onset hearing loss have genetic causes[4,5]. To date, more than 80 genes and more than a hundred genetic loci have been mapped and associated with hereditary hearing loss (http://hereditaryhearingloss.org/).

However, previous epidemiological studies have shown that nearly half of NSHL cases are related to the following genes with recurrent mutations in the Chinese population: *GJB2* (*OMIM*: *121011*), *GJB3* (*OMIM*: *603324*), *SLC26A4* (*OMIM*: *605646*) and the mitochondrial gene *MT-RNR1* (*OMIM*: *561000*) [6–8]. With respect to genetic factors of NSHL in China, *GJB2* variants appear to be the most common (23.37%), followed by variants in the *SLC26A4* (14.74%), *MT-RNR1* (2.56%) and *GJB3*(1.97%) genes[9,10]. Mutations in the *GJB2* gene are the most frequent causes of nonsyndromic autosomal recessive sensorineural hearing loss in most Asian populations, including Han Chinese [6]. Four common mutations (c.235delC, c.299_ 300delAT, c.176_ 191del16, and c.35delG) account for 88.0% of *GJB2* mutant alleles in the Chinese population [11]. A *SLC26A4* mutation is the second most common cause of deafness in China [12]. Mutations in the *SLC26A4* gene are responsible for Pendred syndrome (PDS) and enlarged vestibular aqueduct syndrome (EVA), with hearing loss detected at birth or in early childhood [13,14]. In China, the most common mutation of *SLC26A4* is IVS7-2 A>G, with an allele frequency ranging from 5.13% to 11.06% [10, 15]. The *GJB3* gene, which is related to hereditary NSHL, was first cloned in the Chinese population, and mutations in *GJB3* are associated with progressive hearing loss [7]. The most common mutations in *GJB*3 are c.538C>T and c.547G>A [16]. Although nuclear gene defects constitute the majority of cases of hereditary hearing loss, it has become clear that mutations in mtDNA can also cause NSHL. The best-studied mutations related to aminoglycoside susceptibility are m.1555A>G and m.1494T>C in the *MT-RNR1* gene [17–19]. In a previous study, we showed that the carrier frequency of common mutations of the four above-mentioned genes among 9317 newborns was 3.84% [20], and another study conducted by Zhang showed a higher carrier frequency of 5.52% in northern China [21]. Congenital deafness accounts for the overwhelming majority of the population with prelingual deafness. Because it can affect language capacity, the timing of the detection of hearing impairment is very important. Therefore, early detection, diagnosis, and intervention are necessary for newborns who are susceptible to deafness. Deafness gene screening might identify the cause of deafness at the molecular level and is thus a reliable and effective method for identifying NSHL-associated gene mutation carriers.

At present, different methods are used for the detection of NSHL-associated gene mutations, including classic polymerase chain reaction/restriction enzyme analysis (PCR-RFLP)[22], denaturing high-performance liquid chromatography (DHPLC) [23], matrix-assisted laser desorption/ionization time-of-flight mass spectrometry (MALDI-TOF-MS) [24] and direct sequencing. Such methods present variable challenges; for example, the PCR-RFLP method can detect a limited number of mutations in a single run, making it insufficient for multi-allele detection. Other methods, however, require expensive equipment and personnel with specialized abilities, making them unsuitable for first-line screening in a clinic setting.

In this study, we aimed to develop a more efficient genetic diagnostic assay based on the PCR-RDB technique that could be used as a first-line screening tool for prevalent mutations in the Chinese population. We selected 20 variants of four genes (*GJB2*, *GJB3*, *SLC26A4* and *MT-RNR1*) that are frequently found in the Chinese population and cause NSHL to varying degrees and with differing phenotypes. This platform will serve as a useful and cost-effective first-line screening tool for genetic NSHL in the Chinese population.

## Methods

### Subjects

We collected samples from neonates who were born in Dongguan Children’s Hospital between January 2015 and October 2015. All of the subjects had undergone traditional newborn hearing screening and deafness gene screening by MALDI-TOF mass spectrometry. A total of 225 neonates with at least one of the 20 variants of the *GJB2*, *GJB3*, *SLC26A4* and *MT-RNR1*genes in homozygous, single-heterozygous, or compound-heterozygous forms were recruited. In addition, 30 normal control subjects who passed the traditional newborn hearing screening and did not have any of the 20 variants were also recruited. This study was approved and conducted in accordance with the protocols recommended by the Institutional Medical and Ethics Committee of Dongguan Children’s Hospital. Written informed consent was obtained from the parents or legal guardians of the subjects.

The positive controls used for the development of the assay were clinical blood samples with 18 common NSHL mutations, and the remaining two (c.167delT and c.589G>A) were obtained by site-directed mutagenesis (Shanghai Shanjing Biological Company).

### Genomic DNA extraction

Peripheral whole-blood samples from all of the subjects were obtained in EDTA- containing tubes, and genomic DNA was extracted using the QIAamp DNA Blood Mini kit (Qiagen, Hilden, Germany) according to the instructions provided by the manufacturer. A quantitative estimation of DNA expression was performed by spectrophotometry (Thermo-Fisher Nanodrop, DE, and USA). The genomic DNA products were stored in a freezer at -20°C.

### Design of primers and probes

Ten sets of primers were designed for multiplex polymerase chain reactions (M-PCRs) to amplify the *GJB2*, *GJB3*, *SLC26A4* and *MT-RNR1* genes spanning the 20 mutation sites (Table 1). The 5’ ends of the primers were labeled with biotin. A total of 30 probes, including 10 normal and 20 mutant probes covering the mutation sites, were designed (Table 2). Ten of the normal probes were used as controls for 11 mutant probes, and these included 1226N for c.1226G>A and c.1229C>T. The remaining nine less-common mutations (c.167delT, c.281C>T, c.589G>A, IVS15+5G>A, c.547G>A, c.1975G>C, c.2027T>A, c.1174A>T, and c.2162C>T) had no positive controls. When positive result for any of these nine mutations was obtained, further verification was performed by Sanger sequencing.

**Table 1:** Multiplex PCR primers.

Gene	Mutation sites	Primer sequences	Size (bp)	PCR tube
*GJB2*	c.35delG, c.167delT, c.176_191del16, c.235delC, c.299-300delAT	HL1F:5’- AGAGCAAACCGCCCAGAGTAGAA -3’ HL1R: 5’-GAAGATGACCCGGAAGAAGATGCT -3’	420	Tube I
*SLC26A4*	c.281C>T	HL4F: 5’- GGCTCCCCAAATACCGAGT -3’ HL4R: 5’- TGGTAGCTGGGGAGAAGTG -3’	150	Tube I
	c.589G>A	HL5F: 5’- CAGCTAGAGTCCTGATTGCCA -3’ HL5R: 5’- GCCTTAATAAGTGGGGTCTTGC -3’	250	Tube I
	IVS7-2A>G	HL6F: 5’- CAGCATTATTTGGTTGACA -3’ HL6R: 5’- CCCTTGGGATGGATTTA -3’	304	Tube I
	c.2162C>T, c.2168A>G	HL10F: 5’- AATGCGGGTTCTTTGACGACA -3’ HL10R: 5’- AAATGGAACCTTGACCCTCTTGA -3’	115	Tube I
	c.1174A>T, c.1226G>A, c.1229C>T	HL7F: 5’- GGACCACCACGCAGAGTAG -3’ HL7R: 5’- TGCCATTCCTCGACTTGTTCTC -3’	287	Tube II
	IVS15+5G>A	HL8F: 5’- CAGTCCTATTTTCTATGGCAATGTC -3’ HL8R: 5’- TGCCCTACACAAAGGGAAGAGG -3’	177	Tube II
	c.1975G>C, c.2027T>A	HL9F: 5’- TGCTTACCAAGGAACAGTGTGT -3’ HL9R: 5’- GCCCATGTATTTGCCCTGTTG -3	334	Tube II
*GJB3*	c.538C>T, c.547G>A	HL2F: 5’- GCCCCCTGCCCCAACATCGTG -3’ HL2R: 5’- GTGGCAGCGGCAGGTGGAAGC -3’	200	Tube II
*MTRNR1*	m.1555A>G, m.1494C>T	HL3F: 5’- TTAAGGGTCGAAGGTGGATTTAG -3’ HL3R: 5’- TGGTTTGGCTAAGGTTGTCTGGTA -3’	367	Tube II

**Table 2:** NSHL gene mutation detection probes.

Name of probe	Mutation detection probe (5’→3’)	Name of probe	Normal probe (5’→ 3’)	Detected mutation sites
35M	CGATCCTGGGGGTGTGA	35N	GATCCTGGGGGGTGTGA	*GJB2* c.35delG
176M	CCTGCAGCCAGCTACGATCAC	176N	CAGGCTGCAAGAACGTGTGCTAC	*GJB2* c.176_191del16
235M	CTATGGGCCTGCAGCTG	235N	CTATGGGCCCTGCAGCT	*GJB2* c.235delC
299M	CTACCGGAGACGAGAAGAAGA	299N	CTACCGGAGACATGAGAAGAAG	*GJB2*c.299_300delAT
538M	CTACATTGCCTGACCTACCG	538N	CTACATTGCCCGACCTACC	*GJB3* c.538C>T
1494M	CCGTCACCCTTCTCAAGTATAC	1497N	CCGTCACCCTCCTCAAGTAT	*MT-RNR1* m.1494C>T
1555M	TAGAGGAGGCAAGTCGTAACA	1555N	GAGGAGACAAGTCGTAACATGG	*MT-RNR1* m.1555A>G
IVS7-2M	TTATTTCGGACGATAATTGCT	IVS7-2N	GTTTTATTTCAGACGATAATTGCT	*SLC26A4* IVS7-2A>G
2168M	TGACGGTCCGTGATGCTA	2168N	TGACGGTCCATGATGCTATAC	*SLC26A4* c.2168A>G
1226M	CACTGCTCTTTCCCACACG	1226N/1229N	CTCTTTCCCGCACGGCC	*SLC26A4* c.1226G>A
1229M	GCTCTTTCCCGCATGGC			*SLC26A4* c.1229C>T
167M	GCAGCCAGCTACGATCAC			*GJB2* c.167delT
281M	CGGGAGTTAGTATTGGGC			*SLC26A4* c.281C>T
1174M	GATCAGCTACATCTTCTCAGGA			*SLC26A4* c.1174A>T
1975M	GCCTTCTGCTTGACTGTG			*SLC26A4* c.1975G>C
2162M	ACATTCTTTTTGATGGTCC			*SLC26A4* c.2162C>T
547M	ATTTTCTTCTTGGTAGGTCG			*GJB3* c. 547G>A
589M	CTGACTCTGCTGGTTAGAAT			*SLC26A4* c.589G>A
IVS15+5M	AAGTCCACAGTAAATATTTTATCC			*SLC26A4*IVS15+5G>A
2027M	AGTGAGATCACAGCGGGT			*SLC26A4* c.2027T>A

### Multiplex PCR amplification

Multiplex PCR amplification was performed on an Mx3000p PCR machine (Stratagene, CA, USA). The reactions were conducted in a final volume of 25 μL containing 1×PCR buffer (MgCl_2_Plus), 1×Qiagen Hotstar buffer (Qiagen, Hilden, Germany), 0.2 mmol/L of each dNTP (Promega, CA,USA), 0.2 μmol/L of each of the twenty primers, 0.1 U/μL HotStarTaq DNA polymerase (Qiagen, Hilden, Germany) and 1~2 μg of genomic DNA. The amplificationswere performed in two tubes simultaneously (as detailed in Table 1). The PCR conditions were based on the manufacturer's instructions: pre-denaturation at 95°C for 5 min, 35 cycles of denaturation at 95°C for 30 s, annealing at 55°C for 30 s, and extension at 72°C for 30 s, and a final extension at 72°C for 5 min. The products were subsequently visualized by electrophoresis on a 1.5% agarose gel.

### Reverse dot blot assay

The reverse dot blot (RDB) procedure performed in this study was based on that described by Lappin et al [25]. The carboxyl groups on the surface of the nylon membrane were negatively charged, and oligonucleotide probes were synthesized using a C6-amino-linker on the 5' end of the product, which was positively charged. The probes were fixed onto a Biodyne C nylon membrane (Pall Corporation) after the membrane was activated by incubating with 5% 1-ethyl 3-dimethyl aminopropyl carbodiimide (EDAC) for 30 minutes and then soaking with 0.1M NaOH for 5 minutes.

The target DNA was amplified with 5' biotinylated primers and hybridized to immobilized oligonucleotides on the membrane. All 30 above-mentioned probes were fixed to a Biodyne C membrane (Pall Corporation, NY, USA). The PCR products were heated to at least 95°C and then immediately cooled to 0°C. Each strip and 8mLof hybridization solution A solution consisting of 2×saline sodium citrate (SSC-3 mol/L NaCl and 0.3 mol/Lsodium citrate) with 0.1% SDS (pH 7.4) were pre-heated to 45°C. The denatured PCR products were then added, and the strips were incubated at 45°C for 2 h in a screw-top tube. The strips were subsequently washed with wash solution B (0.5×SSC and 0.1% SDS, pH 7.4) at 45°C for 10 min. Afterward, the strips were transferred to a hybridization solution A-diluted mixture containing 0.125 U/mL streptavidin-horseradish peroxidase conjugate (Roche, Mannheim, Germany) and incubated at room temperature for 30 min. Any excess conjugate was removed through two washes with solution A. The color-developing solution composed of 0.1 mg/mL tetramethylbenzidine dihydrochloride (TMB) substrate (Sigma T8768), 0.015‰ H_2_O_2_ and 0.1 mol/L sodium citrate (pH 5.4) were then added, and the color reaction was developed for 20 min. Blue dots indicated the positive detection. A positive control was performed in each experiment to ensure the reliability of the detection system. All of the samples were analyzed independently by DNA sequencing to confirm the accuracy of the assay.

## Results

### Establishment of the assay

**Fig 1:**
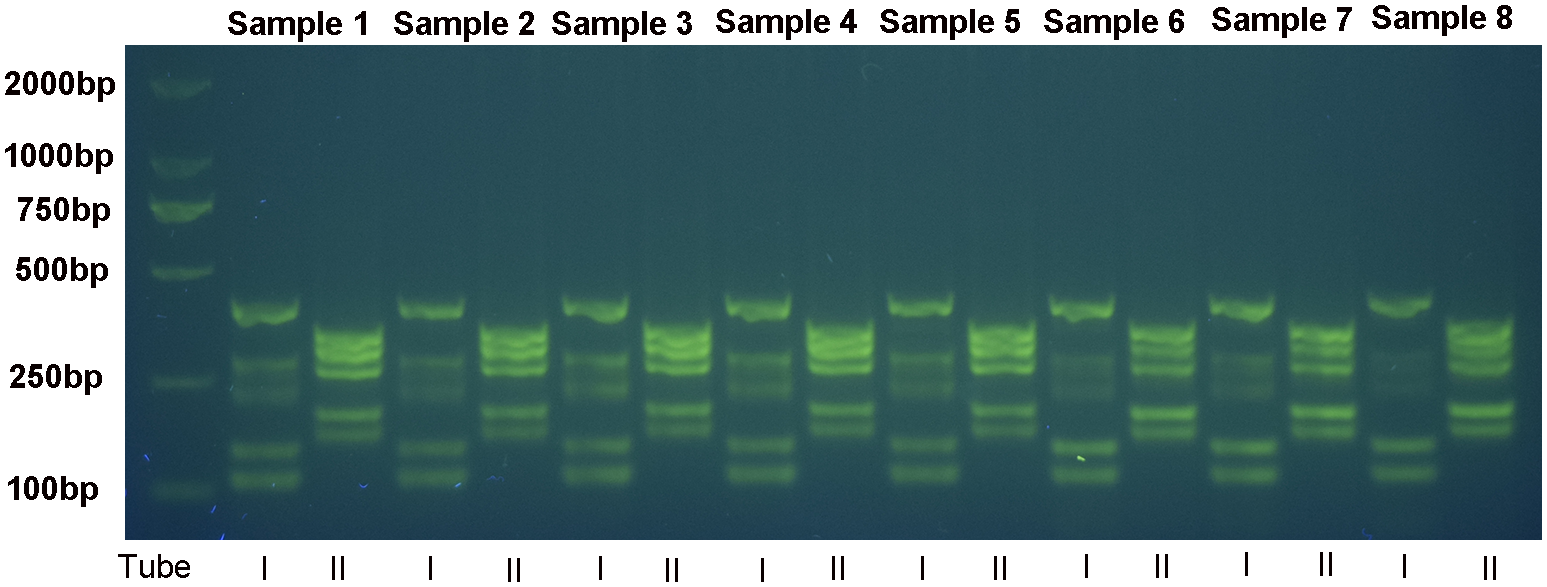
PCR products of eight samples obtained by electrophoresis in a 1.5% agarose gel stained with ethidium bromide. I and II represents PCR products of tube I and II respectively.

The assay was established using DNA samples from 18 subjects with mutations and two plasmid samples expressing the remaining two known mutations, which were introduced through site-directed mutagenesis. The successful electrophoretogram is shown in Fig 1. When analyzing the results, an absence of color development with all or the majority of the control probes served as a invalid results.

**Fig 2:**
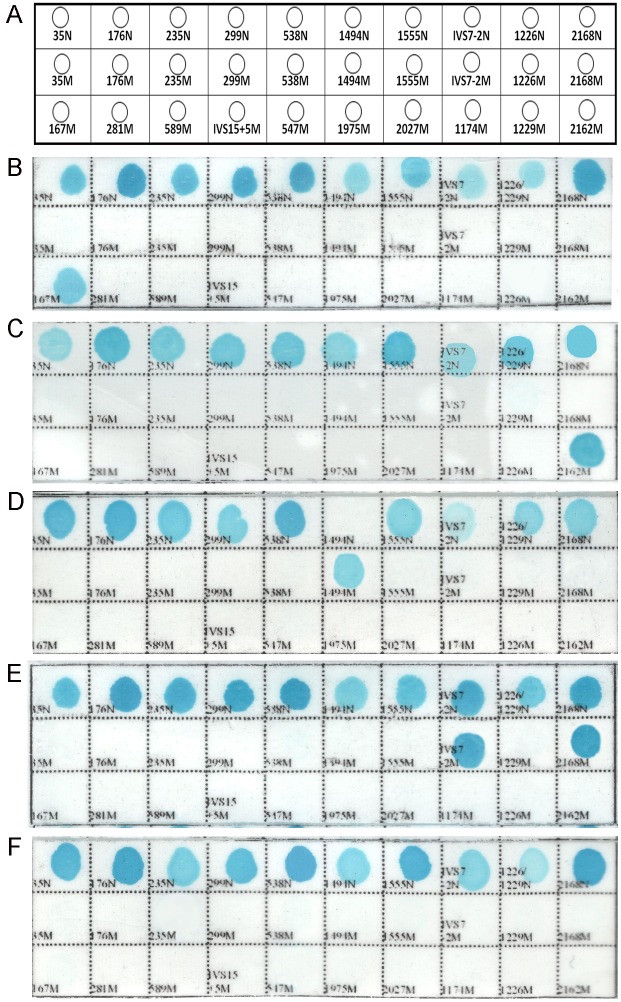
Layout of the probes in the nylon strip designed for the reverse dot blot assay and representative results of the genotyping of 20 NSHL-associated gene mutations using the reverse dot blot assay. N denotes the wild-type probes,and M denotes the mutation probes. (A). Layout of the probes in the nylon strip; (B) c.167delT homozygous or heterozygous mutation; (C) c.2162C>T homozygous or heterozygous mutation; (D) m.1494C>T homoplasmic mitochondrial gene mutation; (E) IVS7-2A>G and c.2168A>Gheterozygous compound mutations; and (F) normal samples.

The assay of the 20 positive control samples tested yielded the expected results (Fig 2 and S1 Fig). The normal DNA samples showed positive blue-colored spots with all of the control probes and negative white-colored spots with the mutation probes. Homozygous DNA samples showed blue-colored spots with the appropriate mutant probes and negative white-colored spots with the corresponding probes. Heterozygous and compound heterozygous DNA samples showed blue-colored spots with one and two mutant probes, respectively, and the corresponding white-colored control-probe spots.

### Validation of the assay

We applied our method to a group of 225 neonates who carried one of 20 deafness gene mutations and 30 normal neonates. All of the samples were subsequently examined by DNA sequencing, and 100% concordance was found between the two methodologies (S2 Fig).

## Discussion

We developed a platform that can be easily performed with common equipment, has a low cost of less than US$3 and can be completed within 6 h. It is an ideal method for smaller clinics and laboratories with limited resources that could allow the front-line detection of NSHL genes without having to resort to direct DNA sequencing. Our results for 255 unrelated subjects showed 100% concordance with the results of independent direct sequencing.

At present, the MassARRAY system combined with an iPLEX assay, which is a high-throughput genotyping technology based on MALDI-TOF mass spectrometry, is the most common deafness gene-detecting method used in clinical settings[26–29]. This MassARRAY system can detect a large amount of variants in multiple samples simultaneously. Nevertheless, the MassARRAY system requires a very expensive platform for the analysis, which limits its use.

Genetic testing can potentially allow an accurate diagnosis of NSHL, and a diagnosis of the genetic etiology will aid the clinical management of patients, including the selection of the most appropriate treatment. Combined newborn hearing screeningand genetic screening of common deafness-related genes will benefit the early detection of infants at high risk of developing delayed hereditary sensorineural deafness or sensitivity to deafness-related drugsas well as the diagnosis of hearing loss in children. The screening of deafness-related genes might effectively prevent the occurrence and development of deafness. The molecular etiology, diagnosis, and effective genetic counseling of the probandare important for the prevention and treatment of deafness.

Because GJB2, GJB3, SLC26A4, and MT-RNR1 are the four most common causative genes of prelingual NSHL in the Chinese population and hotspot mutations of these four genes are included, our assay is expected to cover a substantial portion of prelingual severe-to-profound genetic NSHL in the Chinese population. The restricted targeting reduces the possibility of incidental findings but allows higher coverage at a lower cost compared with genome-wide approaches. This study shows that this simple and inexpensive method can be used for routine molecular diagnosis and potentially for large-scale genetic screening. However, it is possible that the RDB will be affected by the SNP near the mutation. Although this did not occur in the 225 positive samples, we will take test samples in the future study.

We propose that the PCR-RDB assay is a highly accurate, rapid and economical clinical assay that can be potentially used for neonatal hearing screening and personalized medicine. Children with the *MT-RNR1*mutation are recommended stay away from amino glycoside antibiotics to avoid the development of drug-induced hearing loss. Infants with homozygous or compound heterozygous forms of the same autosomal recessive gene may show the symptoms of deafness at birth, and clinical and audiology intervention should be performed as soon as possible. In infants with homozygous or heterozygous mutations in autosomal dominant genes, the mutations can cause deafness, However, some individuals mayexhibit normal hearing at birth and develop delayed deafness, and they should be guided regarding optimizing their listening environment to refrain from developing hearing impairment during their hearing development, e.g., avoidance of head trauma, and timely administration of treatment if their auditory threshold changes.

In conclusion, we developed a new method for the genetic screening of hearing loss and successfully detected hearing loss-related genetic mutations prevalent in the Chinese population in homozygousand heterozygous forms, as validated by Sanger sequencing. This assay is expected to serve as a primary screening tool for genetic hearing loss and could be helpful in gene-based and personalized hearing rehabilitation.

## Supporting information

S1 Fig Results of the genotyping of 20 NSHL-associated gene mutations and normal samples using the reverse dot blot assay. (TIF)

S2 Fig Sequencing results of NSHL-associated gene mutations. (TIF)
