# Supplementary figures and images for: A reverse dot blot assay for the screening of twenty mutations in four genes associated with NSHL in a Chinese population
Source: PLoS One. 2017 May 15;12(5):e0177196. doi: 10.1371/journal.pone.0177196 (PMC5432070; doi:10.1371/journal.pone.0177196)

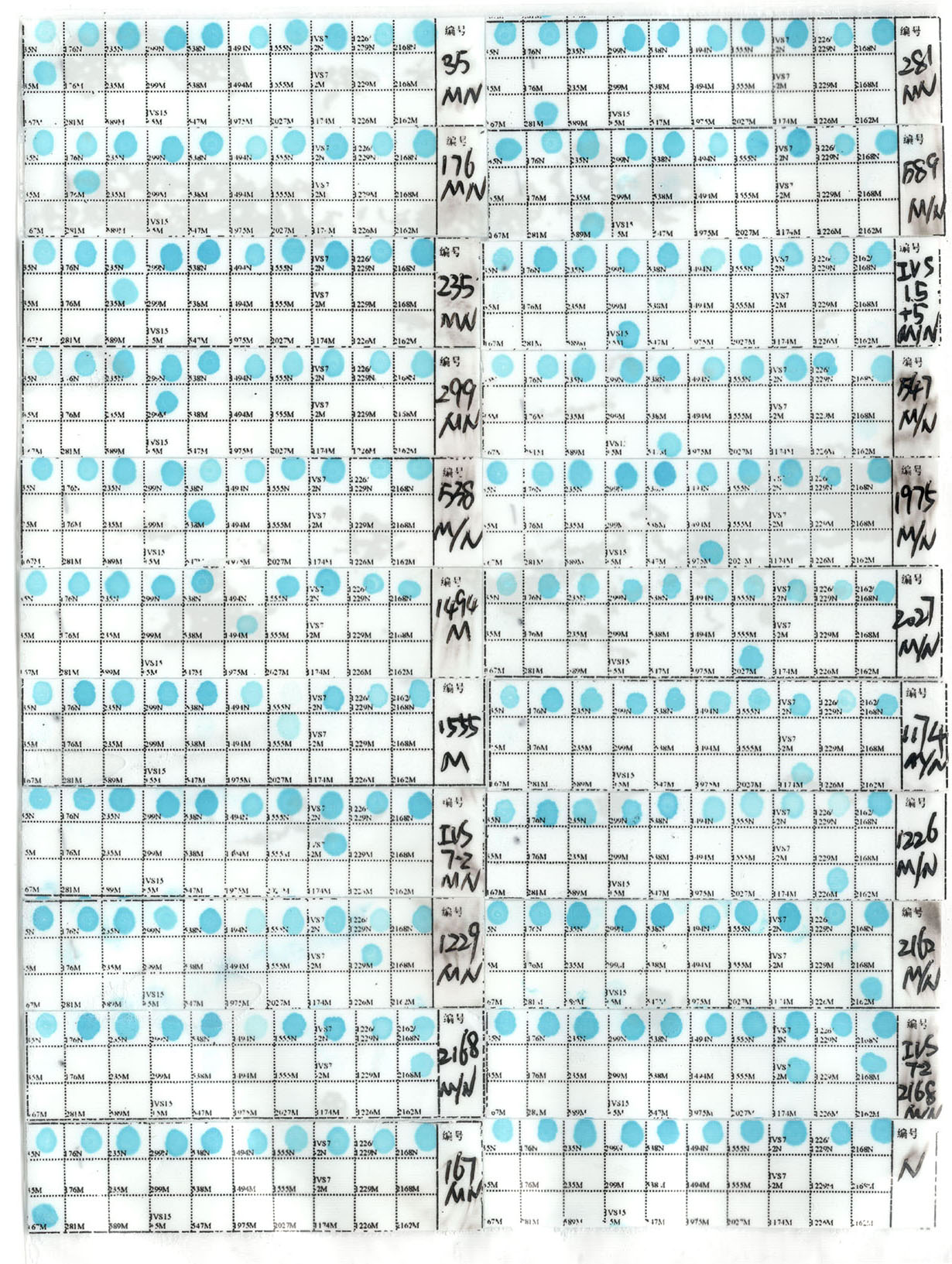

Supplement: S1 Fig — (TIF) [file pone.0177196.s001.tif]

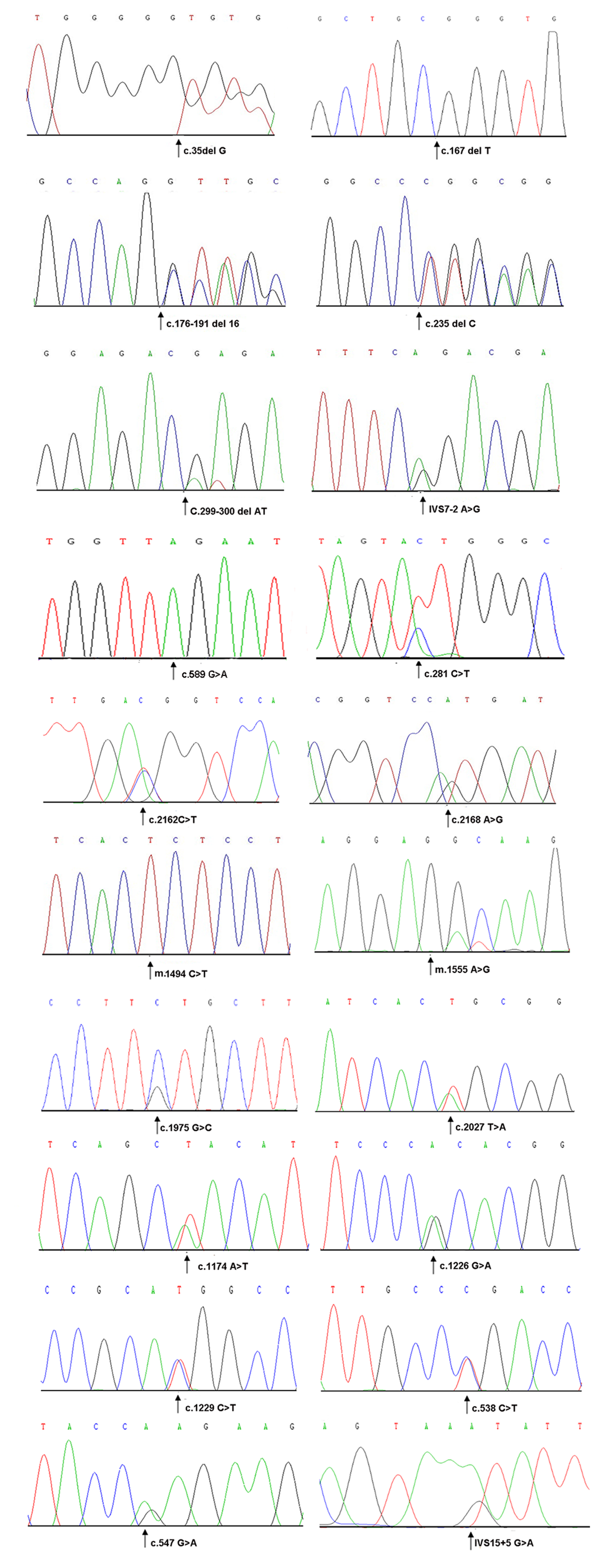

Supplement: S2 Fig — (TIF) [file pone.0177196.s002.tif]
